# Recent Advances in the Role of SLC39A/ZIP Zinc Transporters In Vivo

**DOI:** 10.3390/ijms18122708

**Published:** 2017-12-13

**Authors:** Teruhisa Takagishi, Takafumi Hara, Toshiyuki Fukada

**Affiliations:** 1 Faculty of Pharmaceutical Sciences, Tokushima Bunri University, Tokushima 770-8514, Japan; t.takagishi@ph.bunri-u.ac.jp (T.T.); t-hara@ph.bunri-u.ac.jp (T.H.); 2 Division of Pathology, Department of Oral Diagnostic Sciences, School of Dentistry, Showa University, Tokyo 142-8555, Japan; 3 RIKEN Center for Integrative Medical Sciences, Yokohama, Kanagawa 230-0042, Japan

**Keywords:** zinc transporter, SLC39A/ZIP, zinc signaling, physiology, diseases

## Abstract

Zinc (Zn), which is an essential trace element, is involved in numerous mammalian physiological events; therefore, either a deficiency or excess of Zn impairs cellular machineries and influences physiological events, such as systemic growth, bone homeostasis, skin formation, immune responses, endocrine function, and neuronal function. Zn transporters are thought to mainly contribute to Zn homeostasis within cells and in the whole body. Recent genetic, cellular, and molecular studies of Zn transporters highlight the dynamic role of Zn as a signaling mediator linking several cellular events and signaling pathways. Dysfunction in Zn transporters causes various diseases. This review aims to provide an update of Zn transporters and Zn signaling studies and discusses the remaining questions and future directions by focusing on recent progress in determining the roles of SLC39A/ZIP family members in vivo.

## 1. Introduction

**Figure 1:**
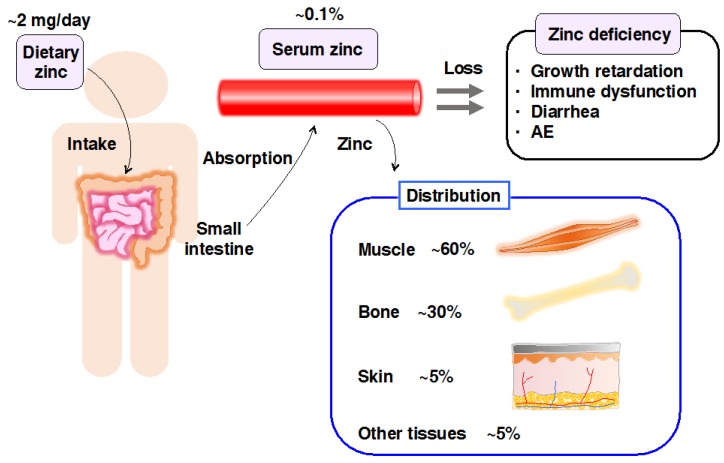
Scheme for Zinc (Zn) storage and distribution in the body. Dietary Zn is absorbed in the small intestine and distributed to the peripheral tissues, including skeletal muscle (60%), bone (30%), skin (5%), and other tissues (5%). Zn deficiency causes various abnormalities in humans and animal models, such as growth retardation, immune dysfunctions, diarrhea, and skin diseases, including acrodermatitis enteropathica (AE).

Zn is an essential micronutrient required for growth, development, immunity, and many other physiological processes. The total amount of Zn in the human body is 2–3 g, with ~60% in the skeletal muscle, ~30% in bone, and ~5% in both, the liver and skin, while the remaining 5% is in other tissues (Figure 1) [1]. Approximately 10% of human proteins may bind to Zn [2], reflecting the indispensability of Zn in numerous physiological processes. Therefore, either a deficiency or excess of Zn is detrimental [1].

Maintenance of intracellular Zn homeostasis mainly depends on two families of Zn transporters: Zrt- and Irt-like proteins (ZIPs), also known as solute carrier family 39A (SLC39A), and Zinc transporters (ZnTs), also known as SLC30A proteins, and metallothioneins (MTs) [1]. ZIPs are known to function in the uptake of Zn across the cytoplasm from the extracellular environment or regulate the release of Zn into the cytosol from intracellular organelles, including the endoplasmic reticulum (ER), mitochondria, and Golgi apparatus; ZnTs acts in the efflux of Zn from the cytoplasm to the extracellular environment or the uptake of Zn into intracellular compartments from the cytosol [1]. Vignesh and Deep describe MTs in detail in this IJMS special issue [3].

ZIPs and ZnTs are involved in many cellular responses, including cytokine- and growth factor-meditated signaling, and the regulation of enzymes, receptors, and transcription factors belonging to cellular signaling pathways [4]. Numerous Zn transporters regulate Zn homeostasis and have crucial functions in physiology; dysfunctions that are caused by mutations result in inherited diseases [1]. Moreover, single-nucleotide polymorphisms, which are related to disease pathology, in each transporter gene have been identified [5,6,7,8]. Thus, impaired Zn transporter function is strongly linked to clinical human diseases, and numerous studies have examined these membrane transporters for their great potential as drug targets.

In this review, we provide the updated information related to Zn transporters, focusing on ZIP family members and their roles in Zn homeostasis, cellular functions, signal transduction, development, and human diseases. We also discuss the remaining questions by reviewing recent progress in studies of Zn transporters and Zn signaling.

## 2. Overview of Mammalian Zrt- and Irt-like Protein (ZIP) Transporters

**Figure 2:**
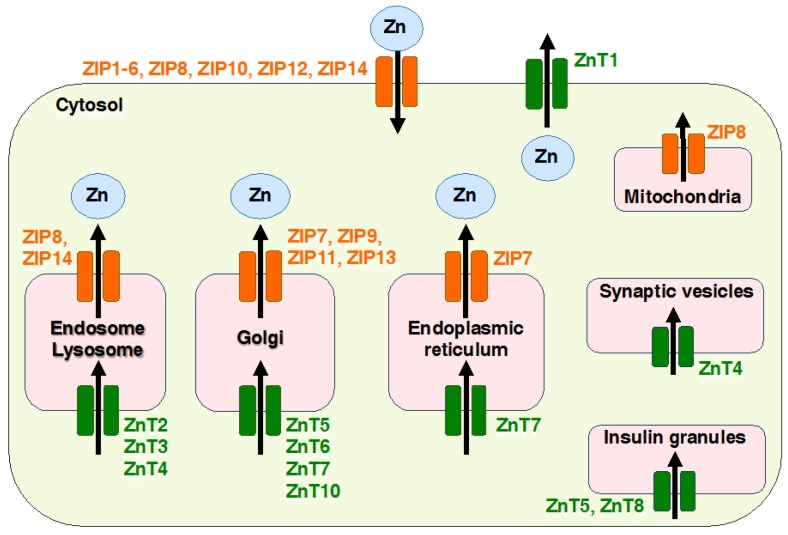
Cellular localization of Zinc transporters (ZnTs) and Zrt- and Irt-like proteins (ZIPs). The diagram shows the localization of ZIPs (orange) and ZnTs (green). The black arrow shows the direction of Zn transport in the plasma membrane and each organelle. ZIPs and ZnTs regulates the flux of Zn ion in the extra- or intra-cellular environment and tightly controls cellular Zn homeostasis in numerous cell types.

Zn regulates a broad range of cellular functions; therefore, the dysregulation of Zn homeostasis causes various abnormalities in mammalian models [1,9,10]. Under physiological conditions, ZnTs reduce the intracellular availability of Zn by accelerating Zn efflux from the cell or into intracellular vesicles, while ZIP transporters import Zn into the cytosol from the extracellular space or intracellular compartments (Figure 2). Some ZIPs and ZnTs have been shown to be involved in the development of human diseases. Moreover, gene deficient (knockout, KO) mouse studies of ZIP and ZnT family members have revealed many unique phenotypes (Table 1), indicating that each Zn transporter-mediated Zn signaling exerts profound effects on non-overlapping molecular events to coordinate physiological conditions. Thus, Zn homeostasis is tightly regulated by the coordination of both transporters. We first provide an overview of all the ZIP transporters, followed by updates of selected ZIP transporters. 

### 2.1. ZIP1

ZIP1 is a prototypic ZIP transporter that transports Zn into the cytosol and is ubiquitously expressed in human tissues [11]. *Zip1*-KO mice are sensitive to dietary Zn deficiency during pregnancy [12]. Previous studies showed that the downregulation of ZIP1 in malignant cells is accompanied by a decrease in Zn [13]. Recently, Furuta et al. observed increased ZIP1 expression in mouse astrocytes under oxidative stress conditions [14]. However, the role of ZIP1-mediated Zn signaling and the relationship between ZIP1 abnormalities and human disease remain unclear.

### 2.2. ZIP2

ZIP2 is known to exist at the plasma membrane in human leukemia cells and functions as an importer of Zn, which increases Zn cellular levels [39]. *Zip2*-KO mice are sensitive to dietary Zn deficiency during pregnancy [15], as are *Zip1*-KO mice [12]. Gene expression analysis revealed high levels of ZIP2 expression in the epidermis, and RNAi knockdown of *ZIP2* gene expression inhibited the differentiation of keratinocytes [40]. Moreover, *Zip2*-KO mice exhibited skin blistering during early embryogenesis [15]. These results indicate that, in the skin, ZIP2 is involved in the differentiation of keratinocytes [40]; thus, ZIP2 is a potential therapeutic target for skin epidermis diseases.

### 2.3. ZIP3

ZIP3 is localized at the plasma membrane in mammary epithelial cells [41], and it functions as an importer of Zn [42]. *Zip3*-KO mice are more likely to show abnormal development during Zn-deficient pregnancy [12,42]. The absence of ZIP3 is evident in early and progressive malignancy; previous studies showed that ZIP3 expression is regulated by Ras-responsive-element-binding-protein (RREB1) in the normal ductal/acinar epithelium [43], indicating that the RREB1/ZIP3 pathway is involved in regulating oncogenesis.

### 2.4. ZIP4

ZIP4 plays an indispensable role in Zn absorption in the small intestine, and it is expressed at the apical membrane of enterocytes [44,45]. Homozygous *Zip4*-KO mice are embryonic lethal during early development, and heterozygous offspring are hypersensitive to Zn deficiency, displaying developmental defects, such as exencephalia, anophthalmia, and growth retardation [16]. Loss-of function mutations in ZIP4 cause acrodermatitis enteropathica (AE), a congenital disease that is characterized by extreme Zn deficiency if it is left untreated without supplemental Zn (OMIM 201100) (Table 2) [46,47]. It has been suggested that dietary Zn is mostly absorbed in the duodenum, ileum, and jejunum by active transport through ZIP4 [48]. However, the molecular mechanisms of dermatitis that is caused by ZIP4 mutation remain unclear. A more recent study investigated whether ZIP4 is cell-autonomously essential for maintaining human epidermal homeostasis [17]. In normal skin, Zn in the basal layer is transported to cells via ZIP4 and sufficiently supplied to Zn-binding proteins, including ΔNp63, which are essential for epidermal differentiation; thus, epidermis-localized ZIP4 has cell-autonomous functions to develop the epidermis. Taken together, ZIP4 has dual roles: ZIP4 increases Zn mass in the body via intestinal ZIP4, and is involved in the development of epidermal tissues by epidermis-localized ZIP4 [17].

### 2.5. ZIP5

ZIP5 is homeostatically expressed in acinar cells and enterocytes, localized to the basolateral surface, and functions as a specific transporter of Zn [53]. A lack of ZIP5 results in Zn accumulation in the liver and failure to accumulate excess Zn in the pancreas [18]. A study of pancreas-specific *Zip5*-KO mice revealed that ZIP5 in pancreatic acinar cells plays a key role in Zn accumulation/retention and protects cells from Zn-induced acute pancreatitis [18]. Although ZIP5 function is required for the survival of mammary gland epithelial cells in culture, homozygous KO mice did not show visible phenotypes. More recently, Feng et al. detected mutations in ZIP5 in patients with high myopia (Table 2) [49]; however, the relationship between ZIP5 and its pathophysiology are not understood. Therefore, ZIP5 may play a unique role in polarized cells by sensing Zn status via serosal-to-mucosal transport of Zn.

### 2.6. ZIP6

ZIP6 localizes to the plasma membrane and functions to import Zn across the cell membrane into cells [54]. The expression of ZIP6 was shown to be associated with estrogen receptor-positive breast cancer, metastatic ability, and cancer progression [20,55,56]. ZIP6 is known to be involved in the epithelial-mesenchymal transition (EMT) and cell migration. During gastrulation in zebrafish, STAT3 transactivates the expression of ZIP6, which promotes nuclear translocation of the transcriptional factor Snail and represses E-cadherin expression [19]. Several studies have revealed similarities and a functional relationship between ZIP6 and ZIP10, suggesting that these Zn transporters interact to conduct biological activities [54,57,58]. It was also shown that ZIP10 is transcriptionally regulated by signal transducer and STAT3 and STAT5, and suppresses apoptosis during the early development of B lymphocytes, and ZIP10 is also overexpressed in human lymphoma [28], as described below. Thus, both, ZIP6 and ZIP10 may be associated with the aggressive behavior of malignant cells, which is regulated by STAT3/5 signaling.

### 2.7. ZIP7

ZIP7 is localized to the Golgi apparatus [59] and ER [60], and plays a critical role in maintaining the intracellular balance of Zn and regulates both cell growth and differentiation pathways involving HER2, EGFR, Src, and IGF1R signaling [1,21,61]. ZIP7 has been shown to be consistently overexpressed in numerous breast cancers with poor prognosis and contributes to the tamoxifen resistance of breast cancer cells [21,62,63].

It has been reported that ZIP7 is involved in growth factor signaling-dependent and/or phosphorylation-mediated signaling pathways [63]. Taylor and colleagues reported that a Zn gate in the ER releases Zn from intracellular stores in response to phosphorylation by casein kinase 2 (CK2), which promotes the activation of tyrosine kinases AKT and ERKs, followed by the regulation of cell migration and proliferation [63]. These findings suggest that ZIP7 acts as a multifunctional protein in regulating a wide range of cellular processes, including ER stress during development and adult tissue homeostasis. In fact, Zn is required for normal ER function, which is supported by the observation that Zn deficiency in the ER lumen causes ER stress [64,65]. In *Drosophila*, Catsup, a member of the ZIP7 protein family, mediates Zn release from the ER and Golgi [66], indicating the possible involvement of ZIP7 in ER functions in vivo.

**Figure 3:**
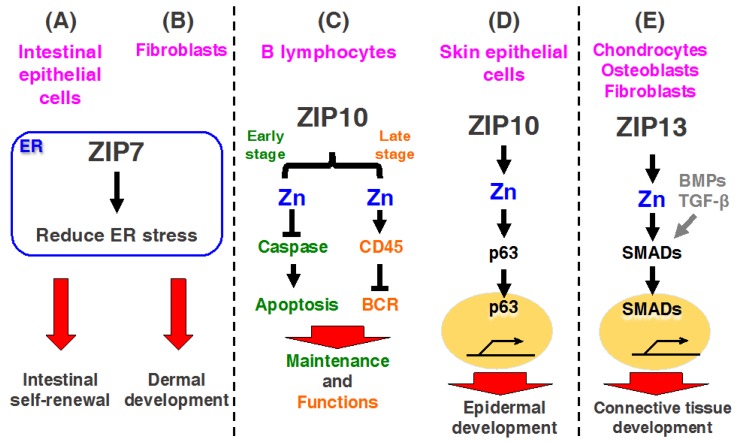
Biological relevance of ZIP7, ZIP10, and ZIP13. (**A**,**B**) ZIP7 is expressed in the endoplasmic reticulum (ER) membrane of various cells including dermal fibroblasts and intestinal epithelial cells, maintains Zn levels in the ER, and contributes to reducing ER stress. (**A**) In intestinal epithelial cells, ZIP7 promotes intestine epithelial self-renewal by resolving the upregulation of ER stress. Therefore, ZIP7 is a new regulator of intestinal epithelium homeostasis by regulating ER function; (**B**) In the dermal fibroblast ER, ZIP7 contributes dermal development. ZIP7 dysfunction induces ER stress caused by Zn-dependent protein disulfide isomerase (PDI) aggregation. PDI aggregation in dermal fibroblast disturbs adequate protein folding, which impairs dermal development; (**C**,**D**) ZIP10 contributes to the development and functions of B cells and skin epidermis; (**C**) ZIP10 inhibits caspase activity in progenitor B cells and promotes B cell development in the early stage (green color). ZIP10 also modulates B cell receptor (BCR) signaling in the late stage (orange color). Thus, ZIP10 is crucially involved in B cell-mediated immunity; (**D**) In skin epithelial cells, ZIP10 up-regulates p63 transactivation, which promotes epidermal and hair follicle development (yellow circle: nucleus). Therefore, the ZIP10-Zn-p63 signaling axis plays an important role in maintaining the skin epidermis; (**E**) ZIP13 is expressed in chondrocytes, osteoblasts, and fibroblasts and contributes to connective tissue development. ZIP13-mediated Zn signaling is required for Smad proteins activation in bone morphogenetic protein (BMP)/transforming growth factor beta (TGF-β) signaling, which regulates connective tissue development.

Recent investigations demonstrated that mice with an intestinal epithelium-specific *Zip7* deletion exhibited ER stress in proliferative progenitor cells, leading to disrupted epithelial proliferation and intestinal stemness (Figure 3A) [23]. Moreover, connective tissue-specific *Zip7*-KO mice exhibited an inhibition of protein disulfide isomerase (PDI), leading to ER dysfunction, which revealed dysgenesis of the dermis and hard connective tissue, including the bone and teeth [22]. Thus, ZIP7 plays an important role in maintaining intestinal epithelial homeostasis and skin dermis development by regulating ER function(s) (Figure 3B) [22,23]. These findings are discussed in detail in subsequent sections.

### 2.8. ZIP8

**Figure 4:**
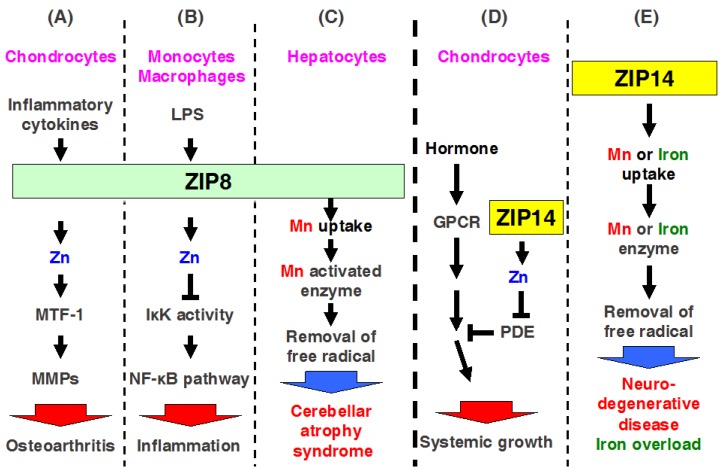
Physiological control by multiple metal transport through ZIP8 and ZIP14. (**A**–**C**) ZIP8 is involved in inflammatory responses and pathophysiology. ZIP8 expression is induced by (**A**) inflammatory cytokines and endotoxin in chondrocyte and (**B**) monocytes and macrophages, respectively. (**A**) In chondrocytes, ZIP8-mediated Zn activates MTF-1 and increases MMP expression, followed by cartilage degeneration of osteoarthritis; (**B**) In monocytes and macrophages, ZIP8-mediated Zn decreases IKKβ activity and NF-κB signaling and promotes inflammatory responses; (**C**) Mn (red) is transported by ZIP8. Loss of function of mutated ZIP8 reduces Mn uptake followed by a decrease in Mn-activated enzymes, resulting in cerebellar atrophy syndrome; (**D**) ZIP14 is required for systemic growth and modulates G protein-coupled receptor signaling by inhibiting hormone-stimulated phosphodiesterase (PDE) in chondrocytes; (**E**) Mn (red) and iron (green) are transported by ZIP14. Loss of function of mutated ZIP14 decreases Mn and iron uptake followed by a decrease in either Mn- or iron -activated enzymes, which results in neurodegenerative disease or iron overload disorders.

ZIP8 is localized to the plasma membrane and apical surface of polarized cells, mitochondria, and lysosomes [67,68]. *Zip8* mRNA expression is a transcriptional target of nuclear factor (NF)-κB, and ZIP8 negatively regulates proinflammatory responses through Zn-mediated downregulation of IκB kinase (IKK) activity, thereby inhibiting NF-κB activity (Figure 4B) [69]. Clinical studies revealed highly elevated serum Zn levels in osteoarthritis (OA) [70]. Kim et al. found that ZIP8 expression is specifically upregulated in OA cartilage of humans and mice, resulting in increased levels of intracellular Zn and the activation of a catabolic cascade by upregulating matrix-degrading enzymes, whereas upregulation of MT1 and MT2 proteins by metal responsive transcription factor (MTF1) forms a negative feedback loop and causes destruction during OA pathogenesis (Figure 4A) [24]. Thus, ZIP8 may be a potent therapeutic target for treating OA.

Interestingly, the mutation of *ZIP8* causes human pathogenesis, including Crohn’s disease and cerebellar atrophy syndrome (Table 2) [50,51]. In fact, ZIP8 possesses higher affinity for Mn than for Zn in cells [67]; moreover, mice with liver-specific *Zip8*-KO mice showed decreased activity of arginase and β-1,4-galactosyltransferase, which are Mn-dependent enzymes (Figure 4C) [71]. Therefore, ZIP8 can regulate both Mn and Zn homeostasis. A loss-of-function mutation in ZIP8 induces the dysfunction of Mn and Zn homeostasis, resulting in human diseases, such as cerebellar atrophy syndrome (Figure 4C).

### 2.9. ZIP9

Previous studies showed that ZIP9 regulates cytosolic Zn levels, resulting in the activation of B cell receptor (BCR) signaling by enhancing Akt and Erk phosphorylation [26]. Notably, ZIP9 is expressed in breast cancer and prostate cancer cell lines [72], and ZIP9 acts as a membrane androgen receptor (mAR) that is independent of nuclear androgen receptors [27]. Testosterone treatment increases intracellular Zn concentrations, thereby upregulating a gene related to apoptosis [72]. These findings suggest that ZIP9 is important for various cellular functions, particularly in some types of cancer cells, where it regulates Zn homeostasis and/or hormone functions.

### 2.10. ZIP10

ZIP10 is mainly localized to the plasma membrane, and it functions as a cell surface Zn importer [28,73]. As described above, ZIP10 forms a functional heteromeric complex with ZIP6 [58]. Recently, ZIP6 and ZIP10 were found to control EMT by inactivating GSK-3 and downregulating E-cadherin in breast cancer cells and renal carcinoma cells [20,74]. ZIP10 is transcriptionally regulated by STAT proteins in early B cells, and is overexpressed in lymphoma, indicating that ZIP10 is involved in the initiation or development of cancers [28]. Interestingly, Bin et al. showed that ZIP10 is required for skin epithelium development, such as the epidermis and hair follicles (Figure 3D) [30]. Together with the requirement of ZIP10 in B cell functions and skin developments, updates on the roles of ZIP10 are described in the next section.

### 2.11. ZIP11

ZIP11 is localized to the nucleus and Golgi apparatus [75,76]. Recently, Martin et al. suggested that ZIP11 plays an important role in the Zn homeostasis required to maintain mucosal integrity, function, and pH within the mouse stomach and colon [75]. Another study suggested that ZIP11 modulates the risk of bladder cancer and renal cell carcinoma [77]. However, the physiological and cellular functions of ZIP11 are not well-defined. 

### 2.12. ZIP12

ZIP12 is highly expressed in human, mouse, and *Xenopus tropicalis* brain tissue [78]. Inactivation of ZIP12 caused developmental arrest and lethality during neurulation in *Xenopus tropicalis* [78]. ZIP12 was shown to play an important role in neuronal differentiation involving the activation of cAMP response element binding protein (CREB) signaling, neurite outgrowth, and tubulin polymerization [78]. A recent study detected ZIP12 expression in pulmonary vascular smooth muscle cells under hypoxic conditions. The inhibition of ZIP12 suppressed cell proliferation and increased intracellular labile Zn in hypoxic-cells [31]. Genetic disruption of ZIP12 in rat attenuates hypoxia-associated pulmonary hypertension in hypoxic environments [31]. Thus, inhibition of ZIP12 may be useful for treating pulmonary hypertension.

Interestingly, a recent study showed that increased ZIP12 expression in the dorsolateral prefrontal cortex causes schizophrenia [79], as described below.

### 2.13. ZIP13

ZIP13 is expressed in hard and connective tissues and, it is mainly localized to the Golgi apparatus [32,33]. Interestingly, a recent study showed that *Drosophila* ZIP13 (dZIP13) transports not only Zn, but also Fe [80]. The amino acid sequence of mammalian ZIP family members determined by the Protein Basic Local Alignment Search Tool (BLASTP) search, revealed that dZIP13 is highly homologous to human ZIP13 [80]. Both the gut and rest of the body exhibited Fe reduction after dZIP13 knockdown, and Fe increase when dZIP13 was overexpressed. dZIP13 affects Fe absorption, as described above, and it is known that dietary Fe absorption is mediated by ferritin in *Drosophila* [81]. Thus, these results suggest that knockdown of dZIP13 inhibits Fe transport into the secretory pathway to be available to ferritin, reducing Fe export from the gut for systemic use, while the overexpression of dZIP13 increases Fe concentrations in the body by facilitating Fe transport into the secretion pathway, making less Fe available in the cytosol of the gut cells. Taken together, dZIP13 potently mediates Fe export to the secretory pathway [80].

Previous studies reported that ZIP13 is involved in bone morphogenetic protein (BMP)/transforming growth factor β (TGF-β)-mediated Smad localization to the nucleus (Figure 3E) [32]. It was demonstrated that bone, tooth, and connective tissues development and systemic growth are impaired in *Zip13*-KO mice, as well as in patients with the loss of functions of ZIP13 proteins [32,82,83]. These patients exhibited significantly decreased white fat mass. Recently, Fukunaka et al. demonstrated that ZIP13-mediated Zn transport plays a critical role in suppressing adipocyte browning by reducing C/EBP-β proteins [84], which are discussed in this issue by Fukunaka and Fujitani. The molecular mechanism of the pathogenesis induced by the mutations is described below.

### 2.14. ZIP14

ZIP14 is localized to the plasma membrane and endosome, and expressed in the small intestine, liver, pancreas, and heart [85,86]. Recent studies have shown that ZIP14 is highly expressed in the various cancers in human including the colorectal cancer, hepatocellular cancer, and prostate cancer [87,88,89,90]. In *Zip14*-KO mice with dwarf body sizes, osteopenia, and impaired skeletal growth, cellular and molecular investigations revealed that ZIP14 modulates G protein-coupled receptor-mediated cAMP-CREB signaling by suppressing basal phosphodiesterase (PDE) activity (Figure 4D) [38]. Moreover, studies with *Zip14*-KO mice have indicated that ZIP14-mediated Zn transports involved in the metabolic endotoxemia, acute and chronic inflammation, intestinal barrier function, hypertrophic adiposity, and impaired glucose metabolism and ER stress [34,35,36,37,91,92]. 

In addition to Zn transportation, ZIP14 has been reported to transport metals such as Fe and Mn in vivo [93,94]. Fe is required for vital metabolic processes in cells; however, excess Fe has toxic effect in cells and can initiate Fe-overload disorders, such as hereditary hemochromatosis, resulting in liver cirrhosis, diabetes, and heart failure [95]. Fe uptake is also known to be regulated by two principle pathways, transferrin (Tf)-Fe via the Tf-receptor (TfR) pathway and nontransferrin-bound Fe (NTBI) through divalent metal transporters, such as DMT1, which is required for intestinal Fe uptake. A previous study using a cell culture system showed that SLC39A14/ZIP14 transport is involved not only in the uptake of Zn, but also in that of Fe in hepatocytes (Figure 4E) [96]. Moreover, a tissue expression array showed that *Zip14* mRNA is ubiquitously expressed at high levels in the liver, pancreas, and heart [86]. Therefore, Jenkitkasemwong et al. evaluated the role of ZIP14 in NTBI uptake in vivo [94]. *Zip14*-KO mice showed decreased ^59^Fe-NTBI uptake in hepatocytes. The authors crossed *Zip14*-KO mice with *Hfe*-KO and *Hfe2*-KO mice to develop an animal model of hemochromatosis in order to determine if ZIP14 is required for tissue Fe accumulation in Fe overload. Analysis of single- or double-KO mice revealed that ZIP14 deficiency in hemochromatotic mice greatly diminished Fe overloading in the liver and prevented Fe deposition in hepatocytes. These findings suggest that ZIP14 is required for NTBI uptake into hepatocytes. Thus, ZIP14 is essential for the developments of hepatic Fe overload in hemochromatosis and for Fe loading of hepatocytes (Figure 4E) [94].

In addition to Zn transportation, ZIP14 has also been reported to transport Fe and manganese (Mn) [93,96]. A recent study demonstrated that ZIP14 is a potent candidate molecule for inducing hemochromatosis [94], and, more recently, ZIP14 was reported to transport Mn in humans [52]. Its loss of function causes similar symptoms as parkinsonism-dystonia with neurodegeneration and hypermanganesemia in childhood (Table 2) [52], indicating that although ZIP14 may be a therapeutic target, further investigations to clarify the molecular basis of ZIP14 are needed, as described below.

## 3. Updates on the Role of ZIP Transporters in Pathophysiology and Human Diseases

In this section, we describe updated information on the role of ZIP transporters, mainly focusing on the pathophysiology. Recently, many studies of ZIP transporters have been conducted, which have improved the understanding of their crucial involvement in physiological events and showing that the loss of their functions causes diseases. Among the ZIP members, we selected ZIP7, ZIP10, ZIP12, ZIP13, and ZIP14 as ZIP transporters, of which investigations in vivo have been remarkably and rapidly progressed, so that advanced information of these molecules are reviewed below.

### 3.1. ZIP7

#### 3.1.1. ZIP7 Contributes to Intestinal Epithelial Homeostasis

Although ZIP7 has attracted much interest in numerous research fields and many studies have been performed in primary cells and cell lines, as described above [21,59,61], the in vivo functions of ZIP7 remained unclear because of the lack of a *Zip7-*KO animal model. A recent investigation demonstrated that ZIP7 is highly expressed in transit-amplifying (TA) cells and Paneth cells at the intestinal crypt [23]. Ohashi et al. generated *Zip7*-conditional KO (*Zip7*-cKO) mice lacking the *Zip7* gene specifically in intestinal epithelium cells [23]. They demonstrated that *Zip7*-cKO mice, which died within a week with the loss of intestinal stem cells and epithelial integrity, showed a loss of the proliferating compartment under increased ER stress. 

ER stress triggers a signaling reaction known as the unfolded protein response (UPR), which plays a crucial role in regulating the proliferation of the intestinal epithelium [97,98]. However, excessive UPR induces ER stress, leading to the activation of apoptosis signaling in the *Zip7*-KO TA cell population. Collectively, these findings suggest that TA cells enhanced UPR signaling and maintained cell proliferation in the lower region of the intestinal crypt. ZIP7 upregulated by UPR signaling maintained Zn homeostasis under ER stress, which promoted epithelial proliferation. This mechanism plays an important role in maintaining intestinal stemness, and it is highly sensitive to the death of neighboring cells induced by ER-stress. Thus, ZIP7 may be a novel regulator of intestinal epithelium homeostasis by maintaining ER function [23]. 

#### 3.1.2. ZIP7 Is Required for Dermal Development in Skin

Skin is the first area that manifests Zn deficiency [99]. However, the molecular mechanisms by which Zn homeostasis affects skin development remain largely unknown. A recent study by Bin et al. further confirmed that ZIP7 is a critical molecule for regulating ER functions in the dermis, and thus it is necessary for proper skin formation [22]. Connective tissue-specific *Zip7-*cKO mice exhibited growth retardation, decreased hair follicles, abnormal incisor teeth, and sunken and down-slanting eyes. Microarray experiments analyzing *ZIP7* expression profiles in human mesenchymal stem cells indicated that the upregulated genes were mainly involved in the response to ER stress, while downregulated genes were mainly involved in cell cycle-related processes and differentiation processes. Moreover, deletion of ZIP7 downregulated PDI activity by increasing ER Zn levels, and induced overexpression of UPR genes [22]. These findings indicate that ZIP7 is a key regulator for resolving ER stress to normalize ER functions; therefore, control of ZIP7 may unlock therapeutic opportunities for overcoming human diseases that are arising from ER dysfunction. 

### 3.2. ZIP10

#### 3.2.1. ZIP10 Is Necessary for the Development and Functioning of B Lymphocytes

Zn deficiency leads to lymphopenia and the attenuation of both cellular and humoral immunity, resulting in an increased susceptibility to various pathogens [100,101]; however, little is known about how Zn regulates immune function. Miyai and Hojyo et al. investigated the expression profile of ZIP transporters and found that ZIP10 was highly expressed in B lymphocytes, particularly in cells in the early stages of B cell development, such as in pro-B cells [28,102]. They evaluated the physiological role of ZIP10 in early B cells by generating B cell-specific KO mice by using *Mb1*-*cre* mice, which exhibited fewer peripheral B cells and decreased pro-B cell survival. *Zip10* ablation in pro-B cells in vitro enhanced the activities of caspase-3, -8, -9, and -12, resulting in increased apoptotic cell death, which was mimicked by chemically chelating intracellular Zn; these negative effects were reversed by Zn supplementation [28] (Figure 3C left). Moreover, they demonstrated that activated STAT3 and STAT5 regulate the ZIP10 expression upon cytokine stimulation [28]. Because it is well-known that JAK-STAT signaling induced by cytokine stimulation controls pro-B cell survival and development [103,104], these findings clearly demonstrate that “the JAK/STAT-ZIP10-Zn signaling axis” is crucial for the survival of pro-B cells during their development [28,102].

Additionally, Hojyo et al. found another role for ZIP10 in late stage B cells by using mice, in which *Zip10* was deficient in antigen-presenting cells; these mice exhibited severely decreases germinal center (GC) formation, which was similar to the abnormalities observed in Zn-deficient mice [29,102]. *Zip10*-cKO late stage B cells showed dramatically decreased proliferation after BCR cross-linking in vitro. BCR signaling is initiated by Lyn, a Src-family protein tyrosine kinase, and Lyn activates Syk, which is involved in the activation of cell proliferation and survival signaling pathways [105,106]. *Zip10*-cKO B cells showed hyperactivated BCR signaling, which reduced cell proliferation (Figure 3C right). Furthermore, CD45R protein tyrosine phosphatase activity was downregulated in *Zip10*-cKO B cells. Thus, the deletion of ZIP10 in late stage B cells led to dysregulated BCR signaling due to reduced CD45R protein tyrosine phosphatase activity and impaired proliferation, as well as decreased GC formation, indicating that the ZIP10-mediated Zn stream is required for proper B cell signaling.

Together, these findings indicate that ZIP10 is indispensable for both, the homeostasis and functioning of B cells; therefore, ZIP10-mediated Zn homeostasis is relevant to B cell-immunity, explaining the importance of Zn in acquired immunity [10].

#### 3.2.2. ZIP10 Is Necessary for Epidermal Homeostasis

The most recent study by Bin et al. revealed that ZIP10 is essential for epidermal formation [30]. As described above, Zn appears to be primarily essential for the differentiation, proliferation, and survival of epidermal keratinocytes in the skin [107]. However, the molecular relationship between Zn homeostasis and cells forming the skin epidermis is not well-understood. ZIP10 was found to be highly expressed in the outer root sheath of hair follicles [30]. Epithelium tissue-specific *Zip10*-cKO mice that were generated by using *Keratine14*-*cre* mice exhibited severe hypoplasia in the stratified epithelia, decreased hair follicles, and thymus atrophy, as the thymus medullae predominantly expresses ZIP10, which plays a role in maintaining this structure [30]. Moreover, the loss of ZIP10 interferes with the functions of p63, a master epidermal regulator containing a DNA-binding domain and Zn binding site [108], indicating the relevance of ZIP10-mediated Zn signaling in p63 function. Thus, ZIP10 plays important roles in epithelial tissue development via, at least in part, the ZIP10-Zn-p63 signaling axis, highlighting the physiological significance of Zn regulation in maintaining the skin epidermis (Figure 3D). These results provide insight for generating new therapeutic approaches by targeting hair follicle-localizing ZIP10. Furthermore, ZIP10-specific agonist may be useful as a trichogenous agent.

### 3.3. ZIP12

#### ZIP12 Contributes to Cortical Functions

It has been reported that ZIP12 is related to the pathogenesis of pulmonary hypertension [31]. However, the relationship between ZIP12 and human disease remains unclear. Scarr et al. demonstrated that in schizophrenia patients, ZIP12 expression is increased in the cortex according to gene expression profiling [79]. Moreover, Zn uptake analysis showed that two variants of ZIP12 have Zn transport functions in cells. However, total cortical Zn levels were not altered in brain tissues from schizophrenia patients, which may be because of changes in Zn homeostasis that are controlled by ZnTs. These results suggest that the increased expression of ZIP12 in brain tissues induces an imbalance in Zn homeostasis, causing the onset of schizophrenia. If ZIP12-specific antagonists are identified, this may be alternative approach for developing more effective drugs for schizophrenia when compared to existing drugs. 

### 3.4. ZIP13

#### Molecular Mechanisms of ZIP13 Pathogenic Mutated Proteins

The first identified genetic disease that was associated with a ZIP family member was AE associated with a mutation in ZIP4 [46,47]; however, the pathophysiological role of ZIP family members except for ZIP4 in human diseases were unknown until 2008. Fukada et al. found that *Zip13*-KO mice show delayed growth and abnormalities in hard and connective tissue development [32], and a loss of function mutations were found in a novel variant of human Ehlers–Danlos syndrome (EDS): Spondylocheirodysplastic Ehlers–Danlos syndrome (SCD-EDS; OMIM 612350), demonstrating the importance of ZIP13 in the development and homeostasis of hard and connective tissues (Table 2) [32,83]. Moreover, they also found that SCD-EDS is attributed to a homozygous loss-of-function mutation in *ZIP13* gene, and genetic analysis showed that the pathogenic mutation was a glycine to aspartic acid substitution at position 64 (G64D) in the ZIP13 protein, which is encoded by *SLC39A13* [32]. Another mutant ZIP13 protein contains a deletion of amino acid residues 162–164 (phenylalanine–eucine–alanine) in *ZIP13*, which was also reported in SCD-EDS patients [83]. Bin et al. revealed that human ZIP13 protein forms a dimer [33,109]. Both mutant ZIP13 proteins are readily degraded by the valosin-containing protein-linked ubiquitin (Ub)-proteasome pathway, presumably by their misfolding during the protein maturation process, resulting in ZIP13 proteins with reduced functions [82]. Thus, ZIP13 mutants are susceptible to Ub-proteasome pathways, and the maintenance of Zn homeostasis via ZIP13 is impaired in cells expressing mutant ZIP13, leading to severe SCD-EDS pathogenesis in *Zip13*-KO mice [33,109] (Figure 3E). However, whether this also occurs in mammals and whether this conclusion explains the onset of SCD-EDS remain unclear. Further studies are required to resolve the complexity of ZIP13-mediated mammalian in vivo physiology and its molecular mechanisms. 

### 3.5. ZIP14

#### ZIP14 Mediates Manganese Homeostasis

Mn is also an essential element for humans and is normally present in various tissues, including the brain, liver, and kidney. Mn imbalance impairs brain functions and causes disorders such as parkinsonism dystonia [110]. Previous studies showed that Mn transport is regulated by several transporter proteins, including DMT1 [111], ferroportin [112], SLC39A8/ZIP8 [71], and SLC30A10/ZnT10 [113], in addition to SLC39A14/ZIP14 [93]. Among these, the molecular details of ZIP14 in Mn transport are currently being examined. The expression and Mn transport function of ZIP14 is regulated by interleukin-6 in human neuroblastoma cells [114]. Recently, Tuschl and colleagues found that loss-of-function mutations in ZIP14 cause hypermanganesemia and progressive parkinsonism [52]. Mutation in *Zip14* by CRISPR/Cas9 genome editing resulted in reduced Mn disturbance in *Zip14*-mutated zebrafish [52], clearly demonstrating the relevant role of ZIP14-mediated Mn homeostasis in maintaining health. 

The most recent reports described the physiological role of ZIP14 in Mn uptake in vivo [93,115]. Interestingly, *Zip14*-KO mice began to show signs of dystonia with a progressive inability to coordinate their motor activities [93], similar to PD-like motor disability in patients with a mutation in ZIP14 [52]. ZIP14 has been shown to be highly expressed in the liver [94]. Therefore, hepatocyte-specific *Zip14*-cKO mice that were generated using *Albumin-cre* mice showed significantly reduced hepatic Mn levels, but not normal levels of Mn in other tissues such as the brain, kidney, and pancreas [93]. In contrast, upon consuming a high-Mn diet, hepatocyte-specific *Zip14*-cKO mice showed increased Mn levels in the serum and brain, but not in the liver. Based on these findings, hepatic ZIP14 regulates the uptake, transport, and storage of Mn. Our results provide insight into Mn homeostasis in humans. Thus, ZIP14 is a potent and major Mn importer in the liver, and recent studies have revealed the clinical effects of disrupting Mn homeostasis (Figure 4E) [52].

## 4. Conclusions and Perspectives

As evident from previous and current studies on the role of Zn in the physiological and pathophysiological events described above, information that is related to the biological relevance of Zn as an essential trace element is accumulating rapidly. Recent studies involving mice and humans suggested that Zn transporters have various physiological functions in tissue development and homeostasis. As described in the sections above, some studies have revealed the relation between the functions of Zn transporters and specific diseases. Despite the current progress in our understanding of the physiological functions of Zn transporters, many questions remain regarding the role of Zn transporters in health and disease.

**Figure 5:**
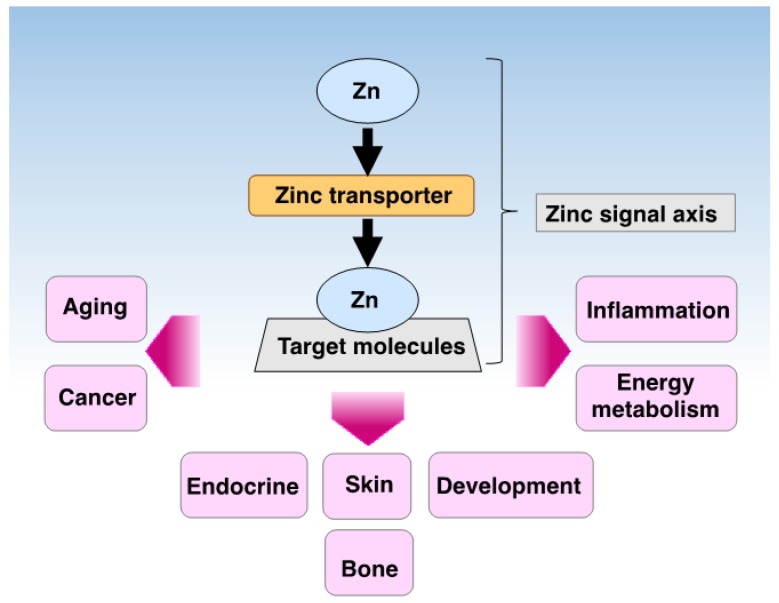
Summary of Zn signal axis in physiology and pathogenesis. Each Zn transporter regulates specific target molecules and cellular responses known as the “Zn-signal axis” [117] which transduces their signals to various physiological processes such as the bone and skin development, endocrine system, and aging. Dysfunction of the Zn signal results in impaired numerous cellular events, leading to various pathophysiological conditions such as inflammation, energy metabolism disorders, and cancer.

Zn transporters are expressed in various tissues and cell types, and they are localized in distinct subcellular compartments; these proteins transport not only Zn, but also Fe, Mn, cadmium, and other trace elements into subcellular compartments, indicating that the functions of Zn transporters are complex and diverse. Thus, studies clarifying the transportation mechanisms, not only of Zn but also of other metal ions, are required to understand the relation between the homeostatic mechanisms of metal ions and various diseases. This information will improve our understanding of the wide-range of functions of Zn ion and its transporters in diverse organisms. These studies may reveal how Zn ion regulates biological functions, and provide information related to its homeostasis (Figure 5) [116].

According to reports on the involvement of Zn transporters in tissue development and homeostasis, the dysfunction of Zn transporters is crucial not only to disease progression, but also to disease onset. Thus, investigating the functions of Zn transporters in tissue development and homeostasis using pluripotent stem cell lines from patients with abnormal Zn-homeostasis is important for addressing these fundamental questions regarding useful disease models. If Zn transporter dysfunctions determine the onset of diseases, modulating their function by specific compounds will provide crucial clues for the development of therapeutic strategies. In addition, no studies have examined the specific compounds that are regulating Zn transporter functions, but such compounds may have great potential for the development of novel therapeutic strategies.

Finally, further intensive research effort to examine Zn transporters would reveal critical molecular mechanisms that are related to mammalian health and disease.

## Figures and Tables

**Table 1:** Physiological properties of SLC39A/ Zrt- and Irt-like protein (ZIP) transporters.

Genes/Proteins	Expression	Subcellular Location	Physiological Functions	Genetic Mutation Study in Mice	References
*Slc39a1*/ZIP1	Ubiquitous	Plasma membrane	Abnormal embryonic development	Knockout (KO)	[12]
*Slc39a2*/ZIP2	Liver, ovary, skin, dendritic cell	Plasma membrane	Abnormal embryonic development	KO	[15]
*Slc39a3*/ZIP3	Widely distributed	Plasma membrane	Abnormal embryonic and T-cell development	KO	[12]
*Slc39a4*/ZIP4	Small intestine, epidermis	Plasma membrane	Embryonic lethality	KO	[16,17]
*Slc39a5*/ZIP5	Small intestine, kidney, pancreas	Plasma membrane	Intestinal Zn excretion; pancreatic Zn accumulation	KO	[18]
*Slc39a6*/ZIP6	Widely distributed	Plasma membrane	Abnormal gonad formation and E-cadherin expression	-	[19,20]
			Glial cell migration in *Drosophila*		
*Slc39a7*/ZIP7	Widely distributed, colon, skin	Endoplasmic reticulum (ER) and Golgi apparatus	Impaired melanin synthesis, fibroblast growth factor receptor (FGFR) and Notch signaling in *Drosophila*	KO	[21,22,23]
			Colon epithelial cell differentiation and proliferation in mouse		
			Skin dermis development		
*Slc39a8*/ZIP8	Widely distributed	Plasma membrane, lysosome	Cdm mouse: Resistance to cadmium-induced testicular damage, embryonic lethality	KO	[24,25]
*Slc39a9*/ZIP9	Widely distributed	Golgi apparatus	Expressed in breast and prostate cancer cell lines	-	[26,27]
			Apoptosis regulation		
*Slc39a10*/ZIP10	Widely distributed, renal cell, carcinoma B cell	Plasma membrane	B cell development and function.	KO	[28,29,30]
			Epidermal development		
			Breast cancer progression		
*Slc39a12*/ZIP12	Brain, pulmonary vascular smooth muscle	Plasma membrane	Neuronal differentiation	KO (Rat)	[31]
			Attenuation of pulmonary hypertension in a hypoxic atmosphere		
*Slc39a13*/ZIP13	Hard and connective tissues	Golgi apparatus, vesicles	Growth retardation, abnormal hard and connective tissue development, and adipocyte browning	KO	[32,33]
			Growth retardation and impaired G protein-coupled receptor (GPCR) signaling		
*Slc39a14*/ZIP14	Widely distributed, liver, bone, and cartilage	Plasma membrane, endosome	Growth retardation, abnormal chondrocyte differentiation	KO	[34,35,36,37,38]
			Adipokineuction		
			Impaired the phosphodiesterase (PDE) activity through GPCR-mediated cAMP-CREB signaling		
			Hypertrophic adiposity		
			Endotoxemia		
			Glucose metabolism		
			Impaired ER stress		

**Table 2:** Hereditary human diseases of SLC39A/ZIP transporters.

Genes/Proteins	Mutation Type	OMIM Gene Locus/Phenotype	Chromosomal Location	Disease	References
*Slc39a4*/ZIP4	Mutation	607059/201100	8q24.3	Acrodermatitis enteropathica (AE)	[17]
*Slc39a5*/ZIP5	Mutation	608730/615946	12q13.3	Nonsymptomatic high myopia	[49]
*Slc39a8*/ZIP8	Mutation, Single nucleotide polymorphism (SNP)	608732/616721	4q24	Cerebellar Atrophy Syndrome, Congenital disorder of glycosylation type II	[50,51]
*Slc39a13*/ZIP13	Mutation	608735/612350	11p11.2	Spondylocheiro dysplastic Ehlers-Danlos syndrome (SCD-EDS)	[32,33]
*Slc39a14*/ZIP14	Mutation	608736/617013	8q21.3	Childhood-onset parkinsonism-dystonia, Hypermanganesemia with dystonia 2	[52]

## Abbreviations

AE	Acrodermatitis enteropathica
EMT	Epithelial mesenchymal transition
ER	Endoplasmic reticulum
KO	Knockout
NTBI	Nontransferrin-bound iron
PDI	Protein disulfide isomerase
SCD-EDS	Spondylocheirodysplastic Ehlers-Danlos syndrome
SLC	Solute carrier
TGF-β	Transforming growth factor beta
UPR	Unfolded protein response
ZIP	Zrt- and Irt-like protein
ZnT	Zn transporter
